# The Weather and the Health of the City

**Published:** 1854-08

**Authors:** 


### THE WEATHER AND THE HEALTH OF THE CITY.

The dog-star rages still. There is no abatement of the intense fervor of the summer. The mercury in Fahrenheit's thermometer is mounting up towards blood-heat, as we write, having risen on the 12th and 13th of the month as high as 98°, and this after a July unprecedented for the intensity of the heat. A drouth is prevailing which threatens all the growing crops, and has already seriously affected the corn. But in the midst of this most unwonted weather the health of the city continues good.

We give on the next page a meteorological table for July, prepared by our friend, Lawrence Young, Esq., the eminent horticulturist and scientific farmer, of Jefferson county.

